# Mental Health Nursing

**DOI:** 10.3390/ijerph17093274

**Published:** 2020-05-08

**Authors:** June Andrews Horowitz

**Affiliations:** College of Nursing, University of Massachusetts Dartmouth, North Dartmouth, MA 02747, USA; jhorowitz@umassd.edu

This Special Issue, *Mental Health Nursing*, provides transdisciplinary readers with a glimpse into the varied interests among researchers in nursing. By design, the articles and subjects featured in this issue were self-selected through submissions and accepted via rigorous peer review. Therefore, these topics do not and are not intended to comprise a representative sample of current mental health nursing research studies. Nonetheless, I anticipate that readers will appreciate the interesting and diverse foci of the featured papers.

The articles reflect multifaceted interests. Notably, topics span the life course from mental health during childhood to older age, although extensive nursing research concerning perinatal, infant and early childhood mental health is not represented. Studies also address a variety of mental health indicators such as depressive symptoms, aggression, and anxiety. Investigations concerning interventions and care plans are featured. Articles also feature experiences and perspectives of healthcare providers and caregivers. Environments include treatment facilities, emergency services, and community settings. Moreover, studies cut across what might be considered traditional specialties in the medical model of health care. Mental health needs and issues are ubiquitous and are not restricted to psychiatric diagnoses or treatment settings, as reflected in the variety of topics found in this Special Issue. 

The breadth and scope of these topics also recall a longstanding nursing framework known as the nursing metaparadigm comprised of four intersecting domains: nursing, person, environment, and health [1]. During more than four decades, this ontology has undergone critique and re-conceptualization. Nevertheless, the interrelations among the domains of nursing, person, environment, and health endure as a guiding framework for nursing practice and research [2]. 

The grouping of articles in this Special Issue reflects this framework. For example, the article, *Identifying the Factors Related to Depressive Symptoms amongst Community Dwelling Older Adults with Mild Cognitive Impairment*, illustrates nursing’s concern with factors and experiences associated with identified symptoms and conditions in a specific context, i.e., community. Another paper, *Encounters with Persons Who Frequently Use Psychiatric Emergency Services: Healthcare Professionals’ Views*, also examines the intersection of the nurses’ (and other providers’) clinical interactions with those persons who repeatedly use mental health emergency services. The study’s focus is situated at the juncture of nurse/person/environment/and health. In addition, the transdisciplinary focus on nurses, assistant nurses or certified nursing assistants, and physicians also speaks to nursing’s longstanding role as core health care team members in collaborative practice with other clinicians.

The issue’s transdisciplinary relevance also extends its value. The topics and methods are highly accessible to many health care and public health readers. Additionally, submissions came from authors from a variety of countries. Thus, the accepted papers reflect diverse perspectives and health care systems globally. Grouping papers under a broad umbrella such as *Mental Health Nursing* is challenging. Yet, regardless of the country of origin, each article addressed one or more aspects and/or intersections of the nursing metaparadigm components of nurse/person/environment/and health. Moreover, when nursing practice was not considered specifically, relevance to clinical nursing practice is clearly evident in the implications. Finally, it is my hope, along with the journal’s editorial staff, that our readers will enjoy this this Special Issue as an accessible window into the diversity of current nursing research internationally, and will spark interest into nursing’s perspective going forward.
